# In Vitro Antibacterial Efficacy of Cetirizine and N-Acetylcysteine Alone and in Combination with Cefalexin on Canine Methicillin-Sensitive and -Resistant *Staphylococcus pseudintermedius*

**DOI:** 10.3390/pathogens15060589

**Published:** 2026-05-29

**Authors:** Jasmin Hawwash, Hilke Oltmanns, Andrea Vanessa Volk, Jessica Meißner

**Affiliations:** 1 Department of Dermatology, Small Animal Hospital, University of Veterinary Medicine Hannover, Foundation, Bünteweg 9, 30559 Hanover, Germany; 2 Department of Pharmacology, Toxicology and Pharmacy, University of Veterinary Medicine Hannover, Foundation, Bünteweg 17, 30559 Hannover, Germany

**Keywords:** microbiology, methicillin-resistant, *Staphylococcus pseudintermedius*, cefalexin, cetirizine, n-acetylcysteine

## Abstract

*Staphylococcus* (*S.*) *pseudintermedius*, as a commensal of the skin and mucosa, leads to a variety of diseases in dogs, most commonly skin and ear infections. The development of methicillin-resistant *S. pseudintermedius* (MRSP) is an emerging risk for animals and humans. The aim of this study was to test cetirizine and N-acetylcysteine as synergistic substances with cephalexin for treating *S. pseudintermedius* infections. Each of the five methicillin-sensitive *S. pseudintermedius* (MSSP) isolates and five MRSP isolates, and one control strain were tested. The minimal inhibitory concentration (MIC) of the substances was tested by broth microdilution assay. In a checkerboard assay, the MIC of cefalexin alone was compared to the MIC of the substances combined. The determined dose reduction index (DRI) shows the influence each substance had on the efficacy of cefalexin. Furthermore, the minimal bactericide concentration (MBC) of N-acetylcysteine (NAC) was identified, and a time kill assay was performed to determine its time-related efficacy on selected isolates. Cetirizine showed no inhibition on bacterial growth or influence on antibiotic efficacy. NAC inhibited bacterial growth at 2 mg/mL. A significant synergistic influence was shown against the MRSP (*p* < 0.001) and MSSP isolates (*p* < 0.01). The MBC of the MSSP isolates and control strain was 12.8 and 25.6 mg/mL for the MRSP isolates. The time kill assay showed that NAC is bactericidal within 120 s at the prior determined MBC concentrations. NAC showed an antibacterial effect alone and a synergistic influence on cefalexin’s antibacterial properties. Thus, NAC shows promising efficacy in treating infections with *S. pseudintermedius*; according to the preliminary study conducted here, this effect may be independent of the resistance profile.

## 1. Introduction

In veterinary medicine, *Staphylococcus* (*S.*) *pseudintermedius* is one of the most frequently detected bacteria in dogs [1,2]. This bacterium is a commensal of the canine skin and mucosal membranes, which can lead to a variety of diseases if the animal’s skin barrier is compromised [3]. As *S. pseudintermedius* is part of the canine skin microbiome, skin and ear infections involving this bacterium are particularly common [4,5,6,7]. Other disease complexes in which *S. pseudintermedius* can be involved include infectious diseases of the eyes, urinary tract, reproductive organs, and respiratory tract [8,9,10,11]. As *S. pseudintermedius* can be involved in so many different diseases, the development of methicillin-resistant *S. pseudintermedius* (MRSP), some strains of which are highly resistant, is of particular concern [12]. Another cause for concern, at the level of the ‘One Health’ concept, is that cases of infections with methicillin-sensitive *S. pseudintermedius* (MSSP) and MRSP are repeatedly diagnosed in humans. In humans, *S. pseudintermedius* is not part of the cutaneous microbiome; its detection is considered an external infection [13]. Since one of the first documented cases of MSSP infections in humans was in 2006 [14], and there are some more recent reports of human MRSP infections [15], the zoonotic potential of *S. pseudintermedius* has become clearer from year to year. Particularly in the field of veterinary medicine, there is a risk of MRSP being transmitted from dogs and cats to humans [16]. With the development of wider resistance patterns, which are highly relevant for both veterinary and human medicine, the pressure to find new solutions to deal with this situation is increasing with each passing year [17]. Since Alexander Fleming discovered penicillin in 1928, antimicrobial agents have been used to treat bacterial infections. It is estimated that tens of millions of lives have been saved to date by using antibiotics [18]. However, in the 21st century, the development of resistances to antibiotics is considered one of the greatest threats to global health [19]. Over the next 25 years, it is estimated that drug-resistant infections could cost several million lives per year worldwide [20]. New antibiotic resistances are emerging faster than new antibiotic agents are developed. Therefore, there is an urgent need for action both to curb the development of resistance and also to research alternative treatment methods [17]. The possible alternatives that have been investigated to date are manifold. One of these alternatives is the use of non-antibiotic agents in treating bacterial infections. Those can have an antibacterial effect themselves or potentiate the efficacy of antibiotic agents through synergism [21]. A checkerboard study by Bruer et al. investigated the effect of antihistamine mepyramine in combination with various antibiotics against *Escherichia coli* isolates in vitro. They detected a synergistic effect between the antibiotics colistin and florfenicol in combination with mepyramine [22]. A more recent study investigated the effect of antihistamine loratadine on methicillin-resistant *S. aureus*. They found that the active ingredient, loratadine, influences the antibiotic oxacillin and leads to a potentiation of the antibacterial effect [23]. As cetirizine is one of the most commonly used and studied antihistamines in dogs, it was chosen for this study [24,25,26,27].

In addition to N-acetylcysteine (NAC)’s ability to break down biofilms formed by bacteria [28], the immediate antibacterial effect of the mucolytic agent has been investigated in various studies in human medicine. As early as 1977, scientists were investigating the antibacterial activity of NAC, showing a good effect of NAC on *Pseudomonas* (*P.*) *aeruginosa* alone and in combination with selected antibiotics [29]. A study conducted almost 40 years later also found an antibacterial effect of NAC against *P. aeruginosa*. This study investigated the combination of NAC with antibiotic ciprofloxacin. The combined therapy led to synergism in 50% of the trials [30]. Several studies on the antibacterial activity of NAC have also been carried out in veterinary medicine, with many of these studies focusing on bacterial pathogens from otitis externa. In 2016, May et al. published a study in which the antibacterial activity of NAC against several isolates of *S. pseudintermedius*, *P. aeruginosa*, and *Corynebacterium* ssp. was demonstrated. The minimal inhibitory concentration (MIC) of the isolates was between 5 and 20 mg/mL [31]. In a follow-up study, the same team under E. R. May investigated the efficacy of NAC in combination with the antibiotics enrofloxacin and gentamicin. Interactions between NAC and enrofloxacin were found to be synergistic in 4.5% of cases, indifferent in 77.3%, and antagonistic in 18.2%. Similarly, interactions between NAC and gentamicin were synergistic in 4.5% of cases, indifferent in 45.5%, and antagonistic in 50% [32]. In 2019, Chan et al. investigated the effect of NAC on otitis-inducing bacterial pathogens. The MIC determined for *P. aeruginosa* was between 2.5 and 5 mg/mL. Chan et al. tested both MSSP and MRSP isolates. The MIC of those were between 2.5 and 10 mg/mL, and there was no clear difference between sensitive and methicillin-resistant isolates [33]. Furthermore, a more recently published veterinary study demonstrated the antibacterial effect of NAC against various isolates of keratitis-causing bacteria in dogs. NAC showed antibacterial efficacy in various concentrations against *P. aeruginosa* (MIC 3.12–6.25 mg/mL), *S. canis* (MIC 1.56–3.12 mg/mL), and *S. pseudintermedius* (MSSP and MRSP; MIC 3.12 mg/mL) [34].

In this study, the use of the non-antibiotic agents cetirizine and NAC, both alone and in combination with the antibiotic cefalexin, was investigated as possible alternative treatment options of MSSP and MRSP infections. Furthermore, the minimal bactericide concentration and the killing time of NAC on selected isolates, as a pilot study, were determined.

## 2. Materials and Methods

### 2.1. Bacterial Isolates

Clinical isolates of *S. pseudintermedius* were collected by the Institute of Microbiology (University of Veterinary Medicine Hannover, Foundation, Germany). The canine isolates derived mainly from superficial skin infections and, in some cases, urine samples. The method matrix-assisted laser desorption/ionization–time-of-flight mass spectrometry (MALDI-TOF score > 2.0) was used to determine the species of the isolates. All isolates were tested for resistance to commonly used antibiotics. Five sensitive and five multidrug-resistant isolates were selected for this study. A control stain (*S. pseudintermedius* DSM 25714) was included.

### 2.2. Minimum Inhibitory Concentration (MIC) Testing

Microdilution, as recommended by the Clinical and Laboratory Standards Institute [35], was chosen as the method to determine the MIC of the eleven isolates. By dissolving and diluting the pure substance NAC (N-acetyl-L-cystein, Sigma-Aldrich Chemie GmbH, Taufkirchen, Germany) in Mueller–Hinton Broth (MHB, Mueller–Hinton–Bouillon, Carl Roth GmbH + Co. KG, Karlsruhe, Germany), different concentrations of the active substance were prepared. NAC was further diluted in MHB to achieve the following stock solutions: 0.5, 1, 1.5, 2, 2.5, 3, 3.5, and 4 mg/mL.

One day prior to testing, the isolate was cultivated on 5% Sheep Blood Columbia Agar (COLUMBIA AGAR WITH SHEEP BLOOD, Oxoid Deutschland GmbH, Wesel, Germany) at 37 °C overnight. By adding colonies from the plate into sterile saline (NaCl 0.9%, B. Braun Medical AG, Sempach, Switzerland) and then adjusting it to 0.5 McFarland standard, the bacterial inoculum was created. The different concentrations were applied to round-bottom 96-well plates (CELLSTAR^®^, 96 Well Cell Culture Plate, sterile, U-bottom, with lid, Greiner-Bio-One GmbH, Frickenhausen, Germany), and 10 µL of the bacterial inoculum was added. A growth control containing 90 µL MHB and 10 µL bacterial suspension, and a negative control per NAC concentration containing 90 µL MHB and 10 µL NAC suspension were also added to the plate. After incubation at 37 °C overnight (16–20 h), the test was evaluated by visual assessment. The first well without button formation corresponded to the MIC.

### 2.3. Checkerboard Assay

To determine whether the combination of two active substances increases the effectiveness of the antibiotic, checkerboard assays were chosen. An increase in effectiveness would be evident by a lower MIC when the substances are combined in comparison to the MIC of the antibiotic alone. One day prior to testing, the isolate was cultivated on 5% Sheep Blood Columbia Agar at 37 °C overnight. By adding colonies from the cultivated isolate into sterile saline and then adjusting it to 0.5 McFarland standard, the bacterial inoculum was created.

The pure substances (Cephalexin Sigma-Aldrich Chemie GmbH, Taufkirchen, Germany; Cetirizine dihydrochloride Alfa Aesar (Thermo Fisher Scientific, Waltham, MA, USA); N-acetyl-L-cystein, Sigma-Aldrich Chemie GmbH, Taufkirchen, Germany) were prepared by dissolving and diluting in MHB to produce the following stock solutions: cefalexin, 0.5 mg/mL; cetirizine, 0.1 mg/mL; and NAC, 200 mg/mL. Depending on the bacterial isolate and their prior determined MIC, different concentrations for cefalexin and cetirizine were used. With the MSSP isolates, cefalexin was diluted to the following concentrations: 0 to 1.5 µg/mL. Cetirizine was diluted to 0 to 6 µg/mL. As for the MRSP isolates, cefalexin was prepared in concentrations of 0 to 80 µg/mL (tests with cetirizine), 0 to 30 µg/mL (tests with NAC), and cetirizine was diluted to 0 to 12 µg/mL. Regardless of the isolate tested, the following NAC concentrations were chosen: 0 to 1.5 mg/mL.

After preparing the different concentrations, each well of a 96-well plate (Greiner-Bio-One GmbH, Frickenhausen, Germany) was filled with an ascending combination of the two substances. Some rows of the plate were used as controls. To start the assay, the bacterial suspension was added to the wells as the last step. After incubation at 37 °C over 24 h, the bacterial growth was determined by absorbance measuring at 570 nm with a microplate reader (MRX Microplate Reader, Dynatech Laboratories, Channel Islands, GB). The first well of the combined substances without bacterial growth corresponds to the MIC combined. The MIC combined was then compared to the MIC alone, as shown below, to determine the dose reduction index (DRI) [36].

$$DRI=\frac{MIC\;alone}{MIC\;combined}$$

A DRI > 1 indicates a synergistic effect of the combined substances. Values < 1 indicate antagonism, while a DRI equal to 1 indicates no influence on the antibiotic by the combined substances. Furthermore, the bacterial growth in per cent was determined according to Bellio [37].

$$\frac{Optical\;densitiy\;\left(OD\right)\;substances\;combined-OD\;medium\;control}{OD\;well\;without\;substance-OD\;medium\;control}\times 100$$

If bacterial growth is reduced by 80% or more, the bacterial growth is considered inhibited.

### 2.4. Minimal Bactericide Concentration (MBC) Testing

In preparation for carrying out the quantitative time-kill assay, the minimum bactericidal concentration (MBC) of the isolates to be tested was determined. The minimum bactericidal concentration corresponds to the concentration at which >99.9% of the bacteria were killed [38]. As an antimicrobial effect of the NAC was demonstrated in the previous tests, the MBC determination and the subsequent time-kill assays as part of a pilot study were exclusively performed for NAC with selected isolates. Since cetirizine does not appear to have such an effect, it was decided not to carry out further tests with this substance.

One day prior to testing, the isolate was spread on sheep blood agar and incubated at 37 °C for 18 to 24 h.

The NAC was weighed out and dissolved in phosphate-buffered saline (PBS), so that a stock solution of 25.6 mg/mL was obtained. This stock solution was diluted in seven further steps by adding 250 µL of the previous solution to 250 µL of PBS. This resulted in a dilution series from 25.6 mg/mL to 0.2 mg/mL. As described above, the bacterial suspension was prepared in PBS. This was diluted twice until 1:100 dilution was obtained.

In a sample vessel, 250 µL of each of the active ingredient dilutions was mixed with 250 µL of the bacterial suspension, which started the experiment. After ten minutes of incubation, 100 µL from each of the sample vessels was added to 900 µL of Dey/Engley neutralizer (Merck KGaA, Darmstadt, Germany). This neutralizer was used to terminate the reaction between the active substance and the bacteria at the selected time points, through its highly bacteriostatic properties [39]. After an incubation time of 5 min, 100 µL was spread onto sheep blood agar plates. For the controls, 900 µL of neutralizer was mixed with 100 µL NAC dilution (negative) and 100 µL bacterial suspension (positive). The plates were incubated at 37 °C for 24 h, after which the colony-forming units (CFUs) were counted. The first plate without growth corresponds to the MBC of the isolate tested.

### 2.5. Time-Kill-Assay

The time-kill assay is an established method for determining the time-dependent effectiveness of an active ingredient, in this case NAC. Two isolates of *S. pseudintermedius* (one sensitive and one methicillin-resistant) and the reference isolate were tested.

In the first step, a stock solution was prepared by dissolving it in PBS to produce a solution with a concentration of 25.6 mg/mL. The bacterial suspension was prepared in PBS as described above. In two further steps, the bacterial suspension was diluted until 1:100 dilution was obtained. 900 µL Dey/Engley neutralization broth was added to sample vessels. Then 1 mL of the bacterial solution was mixed with 1 mL of the NAC stock solution, and a stopwatch was started. After 10, 30, 60, and 120 s, 100 µL was removed and added to the prepared vials with the neutralizer. After 5 min of incubation, the various stopped reactions were diluted again by 1:100 (10 and 30 s) and 1:10 (60 and 120 s). Afterward, 100 µL per time point was spread on sheep blood agar plates. Two control plates were prepared. The plates were incubated at 37 °C for 24 h. On the following day, the CFUs of the different time points were counted. The number of CFUs at 0 s was determined in an additional step by plating out the original bacterial suspension in various concentrations and counting the CFUs.

### 2.6. Data Analysis

The SAS Studio program (Version 9.7, SAS Institute Inc., Cary, NC, USA) was used for the statistical analysis of the checkerboard results. The values determined (DRI) were checked using the sign test and Wilcoxon signed-rank test. A probability error of 5% was considered significant, with values at *p* < 0.05 (*), *p* < 0.01 (**), and *p* < 0.001 (***) being specially labelled. Values at *p* > 0.05 were not considered significant, and thus, they were not labelled separately. The percentage of bacterial growth was calculated using Excel (Excel 2024 Version 2410) and displayed graphically. GraphPad Prism (GraphPad Software 2025) was used for visualizing of the checkerboard assays as heat maps and to create the time-kill curves.

## 3. Results

### 3.1. Microdilution

NAC had an in vitro antimicrobial effect against all tested bacterial isolates. The following table (Table 1) shows the results of microdilution with NAC as MICs of the bacterial isolates (raw data is included as Supplementary Materials). The median of MICs of the methicillin-resistant and -sensitive *S. pseudintermedius* isolates is at a concentration of 2 mg/mL NAC. The results show no significant difference between the MSSP and MRSP isolates. The reference strain (DSM 25714) was tested in comparison. Different microdilutions were carried out with cetirizine. Even at very high concentrations of the active substance (up to 50 µg/mL), no inhibition of bacterial growth by this antihistamine was observed. Therefore, no further microdilutions with cetirizine were carried out.

### 3.2. Checkerboard Assay

The combination of cefalexin with cetirizine showed no change in the DRI of the MRSP isolates. This can be recognized by the fact that a DRI of one was consistently determined in all tests. When the checkerboards with cetirizine were performed on sensitive isolates, no significant change in the DRI was achieved either. In one of the isolates (S-3), the combination of the active substances even led to a median reduction in the DRI, which indicates antagonism (Table 2). The combination of cefalexin with NAC in the MSSP isolates (*p* < 0.01) and in the MRSP isolates (*p* < 0.001) led to a significant increase in the DRI (Table 3).

**Figure 1:**
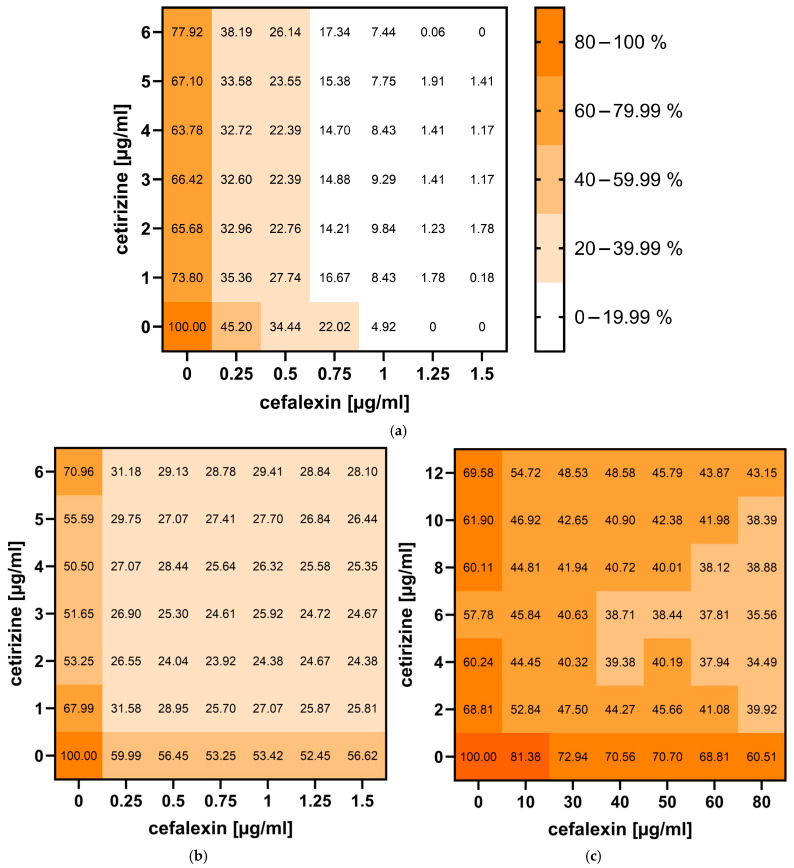
Examples of bacterial growth (in %) in the checkerboard assay with cefalexin and cetirizine (in µg/mL) of isolates (**a**) S-3, (**b**) DSM 25714, and (**c**) MR-2.

As seen from the DRI determined and the bacterial growth (Figure 1) in the tests with cetirizine, it showed no significant anti-staphylococcal effect alone or in combination with cefalexin, even at high concentrations in vitro.

**Figure 2:**
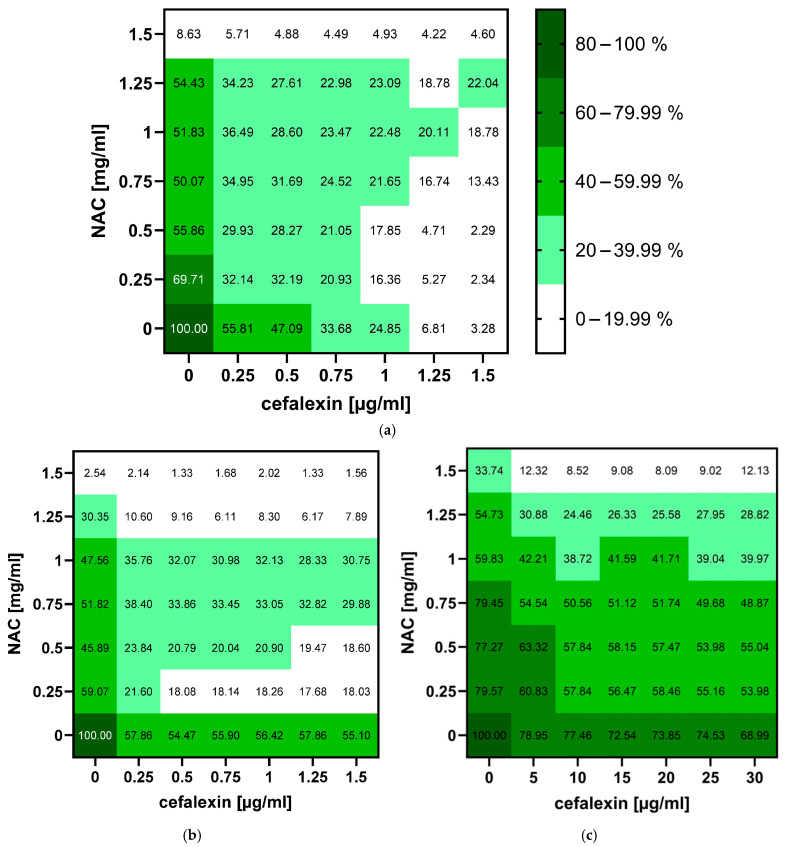
Examples of bacterial growth (in %) in the checkerboard assay with cefalexin (in µg/mL) and NAC (in mg/mL) of isolates (**a**) S-3, (**b**) DSM 25714, and (**c**) MR-2.

In comparison, a clear reduction and inhibition of bacterial growth to <20% was observed in the tests with NAC for the MRSP and MSSP isolates and the reference isolate. However, when testing the MSSP isolates with NAC, it was noticeable that with increasing concentrations of NAC in combination with cefalexin, growth above 20% was observed again in some cases after inhibition had already occurred in the lower concentrations, indicating a dosage-related antagonism. This is shown in Figure 2. The raw data is included as Supplementary Materials.

### 3.3. MBC and Time-Kill-Assay

Table 4 shows the results of the determination of the MBC of two selected isolates and the reference strain DSM 25714 with NAC. The MBCs of the sensitive isolate and the reference strain (DSM 25714) are identical at a NAC concentration of 12.8 mg/mL. The MBC of the methicillin-resistant isolate MR-5 is higher at 25.6 mg/mL.

**Figure 3:**
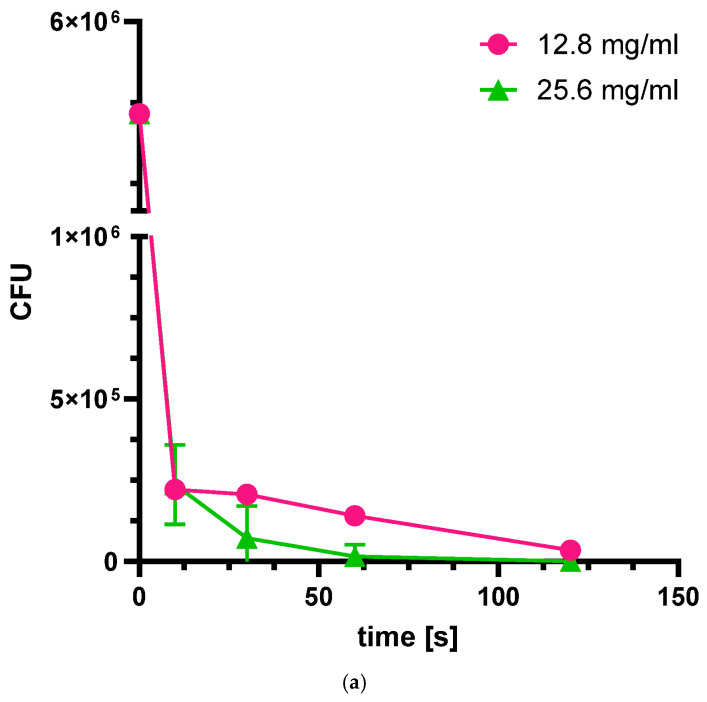

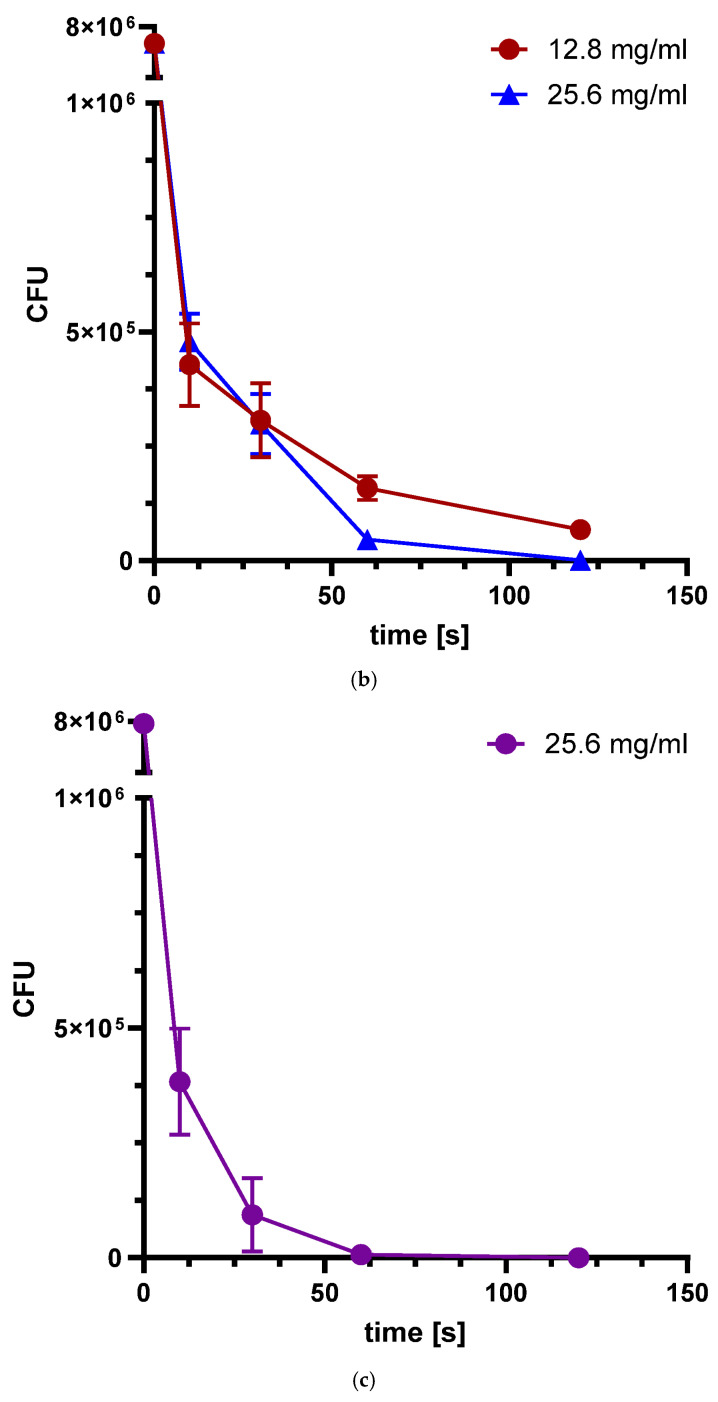
Time-kill curves showing the bactericide effect of NAC on the colony-forming units (CFUs) of (**a**) an MSSP isolate; (**b**) the control strain, DSM 25714; and (**c**) an MRSP isolate.

As the MBC of MR-4 was significantly higher, only the 25.6 mg/mL NAC concentration was tested. By incubating the bacterial suspension in the NAC solution, a significant reduction in CFUs was achieved within 120 s. This is demonstrated in Figure 3, showing the resulting time curves. However, a reduction of >99.9% cannot be achieved with the sensitive isolates and the reference isolate at an active substance concentration of 12.8 mg/mL of NAC, even if a significant reduction in CFUs was achieved overall. A substance is only referred to as bactericidal, if more than 99.9% of the bacteria are killed.

At a concentration of 25.6 mg/mL NAC, an average reduction of 94.5% was already achieved between 0 and 10 s for the MSSP isolate S-5; 92.86% for the reference strain, DSM 25714; and 95.02% for the MRSP isolate MR 4. At 60 s, a reduction of more than 99% was achieved for all three isolates. Finally, at 120 s, an average reduction in CFUs of >99.9% was observed for all three isolates. Thus, this test shows that NAC with a concentration of 25.6 mg/mL has a bactericidal effect on the tested bacteria within 120 s regardless of the resistance level of the tested isolate.

## 4. Discussion

### 4.1. Antibacterial Activity of Cetirizine

In 2011, El-Nakeeb et al. published their in vitro studies on the antibacterial activity of various antihistamines against different multidrug-resistant clinical isolates. In their study, cetirizine showed antibacterial activity against the tested staphylococcal isolates (*S. aureus* and *S. epidermidis*) at a drug concentration of 500–1000 µg/mL [40]. Bruer et al. demonstrated an antibacterial effect at a concentration of 1000 µg/mL of a different antihistamine, mepyramine, against porcine *E. coli* [22,41]. Cetirizine is a commonly used H1-antihistamine in dogs and has a good antihistaminergic effect confirmed in various studies [27,42].

El-Banna et al. hypothesized that properties of antihistamines, such as increased surface activity and, thus, changes in the permeability of the surface membrane of the bacteria, are associated with antibacterial efficacy. The activity on the surface of the membranes appears to increase proportionally with the hydrophobicity of the active ingredient. It can therefore be assumed that hydrophobic antihistamines may have a higher antibacterial activity than those that are hydrophilic, i.e., lipophobic. El-Nakeeb et al. raised a similar hypothesis [40,43]. In comparison, cetirizine belongs to the second-generation antihistamines, which are more hydrophilic and lipophobic substances. This results in less penetration of the blood–brain barrier, which characterizes second-generation antihistamines with subsequent lesser side effects for the patient [44]. If the assumption made by El-Banna is correct, this may explain the lower antibacterial activity of cetirizine found in the studies by El-Banna et al., El-Nakeeb et al., and this present research. However, both El-Banna et al. and El-Nakeeb et al. were nevertheless able to determine an antibacterial effect or potentiation of the antibacterial effect of individual antibiotics at very high concentrations. Other possible mechanisms that could lead to potentiation of the antibiotic’s effect should therefore be considered. These include a potential inhibition of bacterial efflux pumps [43]. However, no antibacterial effect of cetirizine alone and no synergism between cefalexin and cetirizine was detected on the selected clinical isolates in any of the tests carried out at the selected drug concentrations in our study. One explanation for the lack of antibacterial effect could be the choice of substance concentration. Due to the requirement to investigate a potentially clinically applicable approach, significantly lower concentrations were selected in our in vitro study compared to those from the studies by El-Nakeeb et al. and Bruer et al. Concentration ranges were chosen that could be achieved as blood plasma concentrations in dogs according to existing studies. A higher concentration of cetirizine in the trials might have led to evidence of growth inhibition of the bacteria. However, the clinical significance of these results would have been questionable, which is why we decided not to carry out further trials with higher concentrations of cetirizine.

### 4.2. Antibacterial Activity of N-Acetylcysteine (NAC)

In recent years, various scientists from both human and veterinary medicine have demonstrated that NAC is a promising drug with antibacterial activity that can serve as an alternative to traditional antibiotic therapy. The mechanism of action of NAC on bacteria is not yet fully understood; it is likely multifactorial. On the one hand, it is assumed that NAC leads to a competitive inhibition of the uptake of amino acids, such as the amino acid cysteine, by the bacterial cells. Another hypothesis is that the sulfhydryl group within NAC reacts with the bacterial cell proteins [30]. Another important mechanism is the ability of NAC to dissolve the biofilms formed by bacteria by cleaving the disulfide bridges of the mucoproteins [45]. Bacteria encapsulated in a biofilm can have a 10–1000-fold higher resistance to antibiotics [46]. Therefore, the biofilm-dissolving property of NAC, especially in biofilm-forming bacteria and even drug-resistant bacteria, can be a potential enhancement for therapy.

In the present study, NAC was found to have good antibacterial activity against the MSSP and MRSP isolates tested. All tested isolates were sensitive to NAC. An average MIC of 2 mg/mL was found for both the MSSP and MRSP isolates and the reference organism. This is in line with previously conducted and published studies that tested the antibacterial efficacy of NAC against various bacterial species [30,31,33,34]. Regarding later clinical use, study protocol and selected drug concentrations of NAC were applied similarly as for the studies with cetirizine, with the aim to test in a clinically feasible therapeutic range. The literature search for biologically achievable plasma concentrations in dogs yielded only a few results. However, all human and veterinary studies had in common that blood plasma concentrations, even with intravenous administration of high concentrations, are in the µg/mL range [47,48,49,50,51,52]. In a preliminary test, in the course of the present work, no antibacterial activity of NAC could be detected in such a range. In 2010, Zhao and Liu investigated the effect of NAC on *P. aeruginosa* and found that the isolates had an MIC between 10 and 40 mg/mL [30]. May et al. published their study six years after Zhao and Liu, in which they tested *S. pseudintermedius* isolates in addition to *P. aeruginosa*. They found an MIC of all tested isolates between 5 and 20 mg/mL [31]. Chan et al. detected an MIC of MSSP and MRSP isolates between 2.5 and 10 mg/mL [33], and Walter et al. detected MICs of 3.12 mg/mL NAC against 17 MSSP and 3 MRSP isolates [34]. The results of this study are basically consistent with the shown effect of the antibacterial activity of NAC in the earlier mentioned studies above. However, a significantly lower MIC of 2 mg/mL was found for MSSP and MRSP isolates than in the studies mentioned. Our results are most consistent with those of Walter et al. [34]. In contrast to the investigations carried out in this paper and the studies mentioned above, a study from 1977 investigated the effect of NAC on *P. aeruginosa* and found an antibacterial effect at a concentration as low as 20 µg/mL. This was possible because Parry et al. found that the antibacterial efficacy was dependent on the size of the bacterial inoculum treated with the NAC, as well as on the dosage (concentration of active ingredient). Therefore, even low doses could have an antibacterial effect with the appropriate low amount of inoculum [29].

The plasma concentration factor is important to consider because of the potential future clinical use of the results. In comparison to the study with cetirizine, higher concentrations were tested after the initial finding that NAC showed no antibacterial effect at systemically achievable blood plasma concentrations. This is because, with local application, it is possible to treat patients with NAC in higher concentrations than those that can be achieved by oral or intravenous application in the blood. Especially in the field of ophthalmology, NAC has long been used as a topical drug to support the healing of corneal defects. In dogs, it was found that even 20% solutions are tolerated on the eye [53]. The use of NAC, which is already established in ophthalmology, could be a possibility in other areas. NAC could be used topically in different dermatological cases once tolerance has been proven. As early as 2016, Nutall described that biofilms have a major influence on the treatability of otitis and that NAC can be used to dissolve them to make the bacteria more susceptible to antibiotics [54].

Another topic studied in recent years is the effect of NAC on the antibacterial efficacy of various antibiotics. El-Feky et al. published their studies on the combination of NAC with ciprofloxacin against *S. aureus*, *S. epidermidis*, *E. coli*, *Klebsiella pneumoniae*, and *P. aeruginosa*. Their study showed that the effect of ciprofloxacin was potentiated by the addition of NAC at concentrations of 2 and 4 mg/mL [55]. Goswami et al. published that in their combination trials of NAC with various antibiotics against *E. coli*, *Klebsiella pneumoniae*, and *P. aeruginosa*, synergism and antagonism were observed, depending on the antibiotic group. NAC reduced the antibacterial activity of aminoglycosides and fluoroquinolones, while the activity of ß-lactam antibiotics was potentiated. The mechanism of action, which led to different results depending on the antibiotic group, remained unclear [56]. The assumption that NAC in combination with aminoglycosides causes a reduction in antibacterial activity was already made by Parry et al. in 1977 [29], as well as by May et al. [32]. In their combination trials of NAC against typical otitis-inducing bacteria (*S. pseudintermedius*, *P. aeruginosa*, and *Corynebacterium* ssp.), the antibiotics enrofloxacin, a fluoroquinolone, and gentamycin, an aminoglycoside, were tested. The study revealed mainly indifferent and antagonistic effects between antibiotics and NAC [32].

In the present study, a synergism between NAC and cefalexin, a ß-lactam antibiotic, was demonstrated against MSSP and MRSP isolates. In both the MSSP and MRSP isolates, the combination of the active substances led to an increased DRI by a factor up to 6, which corresponds to a 6-fold increase in antibiotic activity. The reduction in the amount of antibiotics required to inhibit bacterial growth was statistically more significant in the MRSP isolates (*p* < 0.001) compared to the analysis of the results with the MSSP isolates (*p* < 0.01), though still statistically significant. These results correspond to the findings of Goswami et al., whose study found an increase in antibacterial activity against various bacteria when combining NAC and ß-lactam antibiotics, such as cefalexin [56].

The potentiation of the antibacterial effect of cefalexin in the present study took place at an active substance concentration of 1 to 1.5 mg/mL NAC.

Various earlier studies and the present study have shown that NAC is a promising alternative for the treatment of bacterial infections. Most of the experiments analyzed in this study determined high concentrations of active substances required for an inhibitory or bactericidal effect of NAC. Clinical use of NAC systemically (via oral or intravenous route) would therefore unlikely be effective according to the available data. However, there are promising results regarding the topical use of NAC, whereby higher concentrations of the active ingredient could be delivered directly to the site of infection.

Thus, time-kill assays were performed to determine how long bacterial isolates must be exposed to NAC to lead to a reduction in their growth. In recent years, various human medical studies have been published on the MBC of NAC. Those tested NAC against various bacteria of relevance to humans, but not against *S. pseudintermedius*. Several studies investigated the influence of NAC on *Enterococcus faecalis* and determined a highly variable MBC of 3.13 to 80 mg/mL [57,58,59,60]. Aslam and Darouiche created time-kill curves, through which they could show that at a concentration of 80 mg/mL NAC a reduction in bacterial growth of >99.9% was observed within 30 min. This was the case for all isolates tested, including methicillin-resistant S. aureus isolates [57].

In the present study, an MBC of 12.8 mg/mL was determined for the selected MSSP isolate and the reference bacterium. The MBC for the selected MRSP isolate was 25.6 mg/mL. Subsequently, time curves were determined for these concentrations regarding the reduction of the CFUs. The time curves showed a reduction in bacterial growth of >99.9% achieved after 120 s with a concentration of 25.6 mg/mL for all isolates (see Figure 3). Although based on a hypothetical model, this rapid efficacy of NAC represents an important finding from this pilot study and may show relevance for potential topical applications where limited contact time is a key consideration. Due to the lower MBCs determined for the sensitive isolates, it was assumed that this concentration of active ingredient is sufficient to achieve an inhibition of bacterial growth by >99.9%. It should be noted that according to the protocol established in this study, the MBC was determined after 10 min of incubation. Thus, the 120 s measured in the time-kill curve may not have been sufficient to observe a bactericidal effect at 16.8 mg/mL NAC. However, even at 16.8 mg/mL there was a strong reduction in bacterial growth of up to 95.02%. In all isolates, there was a very strong reduction in CFUs between time 0 and 30 s.

In comparison to the study by Aslam and Darouiche, the present study showed a significantly shorter required action time of the NAC, despite a lower selected active substance concentration. This could be explained by different bacterial strains being tested, reacting with a lesser sensitivity to NAC [57]. Further conclusions are difficult to draw, as according to the research conducted in this paper, there are currently no studies that have established a time-kill curve of NAC in use against *S. pseudintermedius* isolates. There are some studies with time-kill analyses with various antibiotics against *S. pseudintermedius*. These determined exposure times over several hours to days until a bactericidal effect could be achieved [61,62,63]. Once again, the selected active ingredient concentration is an important factor for the investigations. In the veterinary studies mentioned, antibiotics that are administered systemically and reach a blood plasma concentration in the µg/mL range were investigated. It can be assumed that the active substance concentrations to be achieved in blood plasma, which are in the µg/mL range, have a significantly longer duration of action, compared to the active substance concentration in the mg/mL range selected in our study, before a reduction in bacterial growth of >99.9% can be achieved.

One of the limitations of this study is the relatively small number of isolates tested, which makes it difficult to draw definitive conclusions about the general population. Further studies involving a larger sample size are required in order to substantiate the findings regarding efficacy, particularly in relation to the bacteria’s resistance profile. Additionally, the in vitro nature of the study is another limitation. Results that are conducted in vitro will not fully represent the actual situation in patients in vivo. Furthermore, as this study focused on the direct antibacterial effect, no tests were carried out to assess the NAC’s biofilm-dissolving properties against the isolates. This may limit our understanding of the mechanism underlying the antibacterial activity observed, as it is likely that this property plays an important role.

## 5. Conclusions

Cetirizine showed neither antibacterial activity nor a potentiating effect on cefalexin against MSSP and MRSP isolates at the tested concentrations, consistent with previously published data on other bacterial species. Despite reports of efficacy at very high doses, the clinical applicability of cetirizine as part of antibacterial therapy remains questionable. NAC demonstrated antibacterial activity and potentiated the efficacy of cefalexin against both MSSP and MRSP isolates in vitro, though both effects occurred at concentrations exceeding those achievable through systemic administration in dogs. These findings suggest a potential role for NAC as a topical non-antibiotic adjunct; however, translation to clinical use remains limited and requires further investigations. In particular, the penetration of the active substance into the target tissues should be a focus of future research in order to evaluate its potential clinical application. The additionally observed dose-dependent antagonism in MSSP isolates requires further characterization before combined use can be considered. Future studies should confirm these results with larger sample sizes; further investigate the concentration-dependent antagonism; and evaluate the tolerability, skin accumulation, and required contact times of topically used NAC. Comparative studies assessing NAC against established topical antibiotics such as fusidic acid, gentamicin, and enrofloxacin are needed to define its clinical relevance in veterinary dermatological and otic applications.

## Figures and Tables

**Table 1:** MICs of MSSP isolates; MRSP isolates; and reference strain, DSM 25714, with NAC.

Clinical Isolates	MIC [mg/mL] Median
NAC 0.5–4 mg/mL	
MSSP (n = 5)	2 mg/mL
DSM 25714	1.5 mg/mL
MRSP (n = 5)	2 mg/mL

**Table 2:** MICs of cefalexin in combination with cetirizine for MSSP isolates; MRSP isolates; and the reference strain, DSM 25714.

Isolates	MIC Alone		MIC Combined		DRI	
	Median	Range	Median	Range	Median	Range
MSSP (n = 5)	1	0.75–1.5	1	0.5–1.5	1	0.67–1.33
DSM 25714	1.5	1.5	1.5	1.5	1	1
MRSP (n = 5)	80	80	80	80	1	1

**Table 3:** MIC of cefalexin in combination with NAC for MSSP (n = 5) isolates; MRSP (n = 5) isolates; and the reference strain, DSM 25714. * *p* < 0.01; ** *p* < 0.001.

Isolates	MIC Alone		MIC Combined		DRI	
	Median	Range	Median	Range	Median	Range
MSSP (n = 5)	1.25	0.5–1.5	1.25	0.25–1.5	1	0.67–6 *
DSM 25714	1.5	1.5	0.5	0.25–1.5	3	1–6
MRSP (n = 5)	30	30	5	5–30	6	1–6 **

**Table 4:** MBCs of each MSSP and MRSP isolate and the refence strain, DSM 25714, with NAC.

Isolate	MBC [mg/mL]
Methicillin-sensible *Staphylococcus pseudintermedius*	
S-5	12.8
DSM 25714	12.8
Methicillin-resistant *Staphylococcus pseudintermedius*	
MR-4	25.6

## Data Availability

The dataset used and analyzed during the current study are available from the corresponding author upon reasonable request.

## Supplementary Materials

The following supporting information can be downloaded at https://www.mdpi.com/article/10.3390/pathogens15060589/s1.

## Abbreviations

The following abbreviations are used in this manuscript:

CFUs	Colony-forming units
DRI	Dose-reduction index
MALDI-TOF	Matrix-assisted laser desorption/ionization–time-of-flight mass spectrometry
MBC	Minimal bactericidal concentration
MHB	Mueller–Hinton Broth
MIC	Minimal inhibitory concentration
MRSP	Methicillin-resistant *Staphylococcus pseudintermedius*
MSSP	Methicillin-sensitive *Staphylococcus pseudintermedius*
NAC	N-acetylcysteine
*P.*	*Pseudomonas*
PBS	Phosphate-buffered solution
*S.*	*Staphylococcus*
